# Coexistence of Intracranial Aneurysm and Pituitary Adenoma: A Case Report and Literature Review

**DOI:** 10.7759/cureus.103273

**Published:** 2026-02-09

**Authors:** Javiera Gassmann, Patricio Álvarez, Roberto González, María Consuelo Guzman, Felipe Maldonado

**Affiliations:** 1 Department of Anesthesiology and Perioperative Medicine, Hospital Clínico de la Universidad de Chile, Santiago, CHL; 2 Department of Neurosurgery, Hospital Clínico de la Universidad de Chile, Santiago, CHL

**Keywords:** aneurysmal subarachnoid haemorrhage, brain tumor, complicated subarachnoid hemorrhage, neuro anesthesia, pituitary adenoma, pituitary adenoma management, transsphenoidal neurosurgery

## Abstract

Unrecognized intracranial aneurysms pose a rare but catastrophic risk during transsphenoidal surgery. Pituitary adenomas are among the brain tumors most frequently reported to coexist with intracranial aneurysms, yet routine preoperative aneurysm screening is not universally recommended. We report a 73-year-old woman who underwent elective transsphenoidal resection for recurrent pituitary adenoma and developed sudden hemodynamic instability and massive epistaxis intraoperatively, followed by an abrupt reduction in electroencephalographic activity with increased suppression on processed EEG monitoring despite unchanged anesthetic targets. Computed tomography angiography demonstrated a ruptured pseudoaneurysm involving the anterior communicating artery complex with extensive subarachnoid and intraventricular hemorrhage and hydrocephalus. Despite intensive surgical and critical care, the hemorrhage progressed with secondary cerebral injury. After discussion with the family, life-sustaining measures were withdrawn, and the patient died. This case underscores that the rupture of an unrecognized aneurysm during elective transsphenoidal surgery, although uncommon, can be rapidly fatal and difficult to control intraoperatively, and supports careful preoperative cerebrovascular assessment, particularly in patients with recurrent or anatomically complex sellar disease.

## Introduction

An increasing number of reports describe an association between brain tumors and intracranial aneurysms, with pituitary adenomas among the tumor types most frequently linked to aneurysm formation [1]. Although the precise mechanism by which pituitary adenomas may contribute to aneurysm development remains unclear, several studies have for years suggested that intracranial aneurysms are more prevalent in patients with pituitary adenomas than in the general population [2-4]. Because routine aneurysm screening is not universally recommended for all patients with pituitary adenomas, we present the case of a patient with a recurrent pituitary adenoma who suffered a catastrophic subarachnoid hemorrhage due to rupture of an undiagnosed intracranial aneurysm during transsphenoidal surgery. Additionally, we provide a literature review on the coexistence of pituitary tumors and intracranial aneurysms, addressing their epidemiology, risk factors, pathophysiology, diagnosis, and management.

## Case presentation

**Figure 1:**
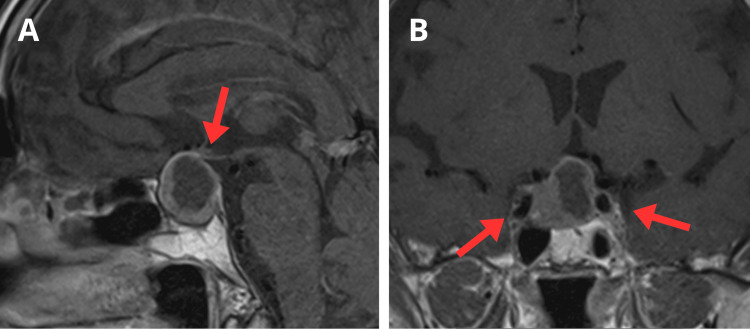
Preoperative magnetic resonance imaging of the pituitary gland Sagittal T1-weighted gadolinium-enhanced MRI (A) and coronal MRI (B) demonstrating a cystic pituitary macroadenoma, suggestive of apoplexy. The tumor exhibits suprasellar extension, compressing the optic chiasm (A), and invasion of the right cavernous sinus (Knosp grade 2) (B).

A 73-year-old woman with hypertension, hypothyroidism, and dyslipidemia, and a history of a nonfunctioning pituitary macroadenoma, resected twice via transsphenoidal surgery at an outside institution (11 and six years earlier), presented with an asymptomatic residual pituitary tumor. Operative and histopathology reports from prior procedures were unavailable. Magnetic resonance imaging (MRI) demonstrated a mass measuring 18 mm craniocaudally, 19 mm transversely, and 19 mm anteroposteriorly, consistent with a cystic pituitary macroadenoma suggestive of apoplexy, with no additional abnormalities reported by neuroradiology and no prior imaging available for comparison (Figure 1).

The patient denied any significant surgical or anesthetic complications during her previous operations. Cardiovascular evaluation revealed well-controlled hypertension without symptoms of coronary artery disease or heart failure, and a Duke Activity Status Index (DASI) score of 18.95. Given that the tumor size precluded stereotactic radiosurgery and that radiotherapy was associated with a high risk of sequelae, an elective transsphenoidal resection of the residual tumor was planned.

An effect-site target-controlled infusion (TCI) of propofol and remifentanil for total intravenous anesthesia was initiated and guided using the SedLine^Ⓡ^ density spectral array (Masimo Corporation, California, US). Initially, the otolaryngology team accessed the sphenoid sinus endoscopically via a binasal approach to provide optimal visualization and exposure of the surgical field. The neurosurgical team then proceeded with tumor resection following standard protocol. At the beginning of the neurosurgical stage, endoscopic inspection revealed no prior opening of the sella turcica; instead, a defect in the anterior sellar floor with cerebrospinal fluid (CSF) egress was observed. The opening was enlarged, briefly exposing the cerebral parenchyma, prompting immediate closure. A dural repair was performed with an autologous fat graft and Surgicel (Ethicon, Ohio, US), after which the sellar floor was reconstructed, and a new opening in the bony floor was created with a drill and Kerrison rongeurs (Aesculap instruments, B. Braun, Tuttlingen, Germany). A cruciate dural incision was then made, exposing a soft, violaceous cystic-solid tumor.

Tumor debulking was performed by evacuating all four quadrants with curettes and dissectors, after which descent of the diaphragma sellae was noted. The cavity was thoroughly irrigated, and gross-total resection was confirmed macroscopically. Closure was initiated with an autologous fat graft and Surgicel; however, during graft placement, brisk bleeding from the meningeal borders of the sellar region occurred. Hemostasis was attempted using Surgiflo (Ethicon, Ohio, US) and Surgicel packing.

**Figure 2:**
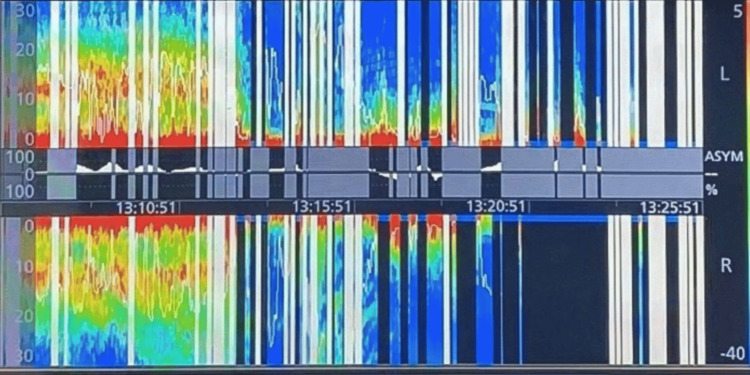
Anesthetic depth monitoring record The SedLine^Ⓡ^ (Masimo Corporation, California, US) spectrogram displayed the patient's electroencephalographic power changes across various frequency bands. The characteristic high alpha and delta power propofol/remifentanil TCI pattern abruptly shifted to a reduction in alpha band power, an increase in delta band power, and subsequent episodes of suppression, represented by black lines with blue dots on the density spectral array.

During graft placement, the patient developed a spontaneous sinus pause accompanied by hypotension, which responded to 0.5 mg of intravenous atropine. Shortly thereafter, a sudden rise in blood pressure was followed by massive epistaxis and a drop in hemoglobin from 11.6 to 9.5 g/dL. Epinephrine-soaked nasal packing and tranexamic acid were administered, achieving temporary hemorrhage control. Upon reinspection of the sellar cavity, apparent hemostasis was confirmed. Notably, the SedLine® monitor demonstrated an abrupt reduction in electroencephalographic activity with an increased suppression ratio, despite no changes in the effect-site targets for propofol or remifentanil (Figure 2).

**Figure 3:**
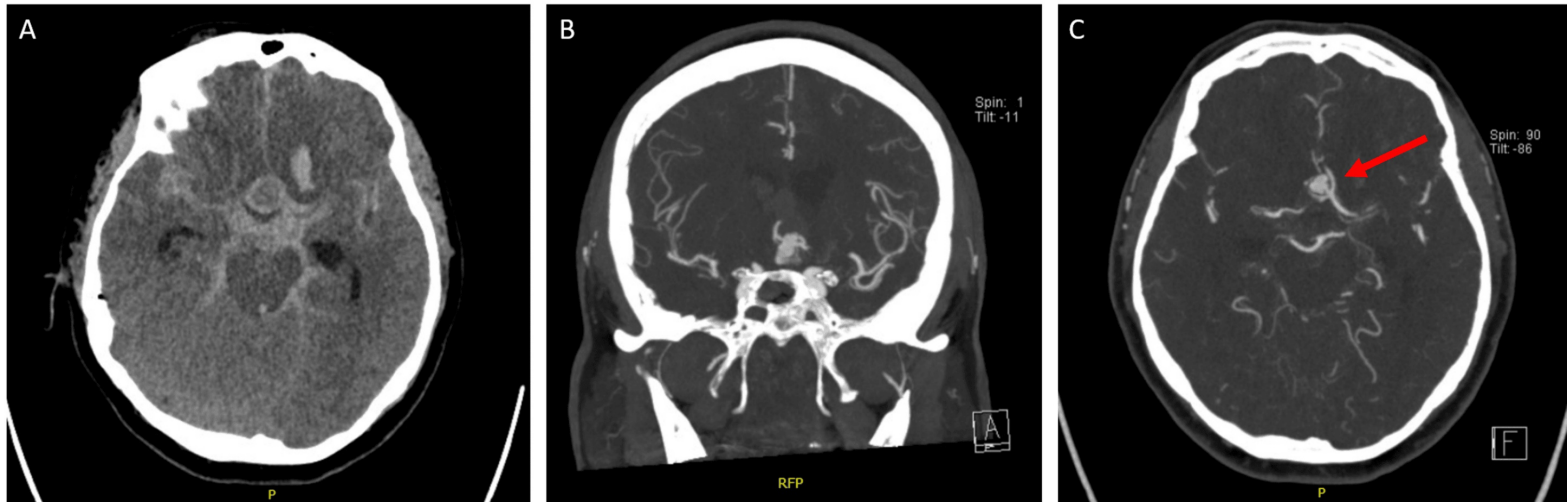
Images of the patient's multiplanar cerebral aneurysm and subarachnoid hemorrhage The images represent different projections from a cerebral computed tomography angiography (CTA) performed on the patient. The axial view (A) reveals an extensive subarachnoid hemorrhage with a potential aneurysmal lesion associated with the anterior communicating artery complex. The coronal view (B) further characterizes an aneurysm or pseudoaneurysm about the same arterial structure. Lastly, the axial image in (C) presents a suggestive view of an aneurysm or pseudoaneurysm with the location of the lesion indicated by the arrow.

Given the absence of neurologic recovery and the concerning intraoperative events, the team proceeded directly to computed tomography angiography while the patient remained intubated, rather than attempting emergence from anesthesia. Imaging revealed a ruptured pseudoaneurysm involving the anterior communicating artery complex, with extensive subarachnoid hemorrhage, intraventricular hemorrhage, and hydrocephalus (Figure 3).

Digital subtraction angiography and endovascular aneurysm management were considered; however, definitive endovascular treatment was deferred, and urgent cerebrospinal fluid diversion was prioritized. An external ventricular drain (EVD) was placed, with an opening intracranial pressure of 33.9 cmH_2_O. Osmotherapy and hemodynamic management were subsequently adjusted to optimize neuroprotection.

After EVD placement, anisocoria was detected, and a front-temporoparietal decompressive craniectomy to manage an evolving cerebral herniation resulted in superior sagittal sinus hemorrhage with profuse bleeding. Severe refractory hemodynamic instability developed, evolving into cardiopulmonary arrest with pulseless electrical activity. Spontaneous circulation was restored after 60 seconds of resuscitation maneuvers (chest compressions), high-dose vasoactive drugs, and transfusion of blood products. Sinus bleeding was controlled with direct compression and application of a topical hemostatic agent. After complete evacuation of the subdural hematoma, the patient was transferred to the intensive care unit (ICU) for further stabilization and deferred aneurysm exclusion.

**Figure 4:**
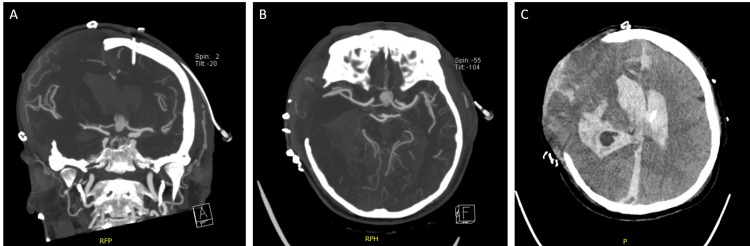
Brain computed tomography taken 12 hours after decompressive craniectomy A) and B) show coronal and axial views of a cerebral computed tomography angiography, respectively, the aneurysm associated with the anterior communicating artery, significant cerebral edema, and a right-sided craniectomy; C) Axial view shows the subarachnoid hemorrhage, a large hemoventricle, hypodensity, and significant cerebral edema and herniation at the site of the right craniectomy, with a ventricular catheter positioned in the left lateral ventricle.

The patient remained sedated under neuroprotection in the ICU with high requirements for vasoactive drugs. Follow-up computed tomography angiography showed significant aneurysmal formation suggestive of a pseudoaneurysm related to the anterior communicating complex with a new posterior component of approximately 7 mm, increased intraventricular and subarachnoid blood; persistent brain parenchyma herniation due to craniectomy; and ischemia in the right cerebral hemisphere, with cerebral and cerebellar edema (Figure 4).

Following consultation with the family, life-sustaining measures were withdrawn. The family declined an autopsy. The patient died shortly thereafter in the presence of family members.

## Discussion

Here, we report the rupture of an unrecognized intracranial aneurysm during elective surgery for a pituitary tumor. We highlight the surgical and anesthetic findings and the management challenges that arise in this type of emergency. Because cases with unfavorable outcomes are less frequently reported, we provide detailed descriptions to support learning and inform decision-making at other centers.

Epidemiology

The association between pituitary tumors and intracranial aneurysms has been reported for several decades [4], although whether this relationship is causal or merely coincidental remains debated [5]. Improvements in neuroimaging resolution and the increasing use of preoperative cerebrovascular evaluation likely contribute to a higher number of reported cases [6,7].

Offret and Aron, later followed by Pia et al., first suggested an association between intracranial aneurysms and brain tumors, reporting an approximately two-fold higher incidence in meningiomas and pituitary adenomas compared with the general population [4,8]. Jakubowski and Kendall reviewed angiograms from 150 pituitary adenomas and 33 craniopharyngiomas and identified 11 incidental anterior-circulation aneurysms, most arising from the cavernous or supraclinoid internal carotid artery and the anterior cerebral complex. Growth hormone-secreting adenomas showed a higher prevalence than chromophobe adenomas (13.8% vs 5.1%) [9]. Wakai et al. reported a higher prevalence of anterior-circulation intracranial aneurysms in patients with pituitary adenomas than in those with other brain tumors (7.4% vs 1.1%) [10].

Later studies assessed this association. In a series of 800 patients with pituitary adenoma, Oh et al. reported coexisting intracranial aneurysms in 2.3% of cases; older age and cavernous sinus invasion were independent correlates, and acromegaly was associated with a higher aneurysm prevalence and higher risk of subarachnoid hemorrhage [11]. A meta-analysis of 68 studies reported a prevalence ratio of 2.0 (95% CI 0.9-4.6) for unruptured intracranial aneurysms in patients with pituitary adenomas. However, the sex- and age-adjusted prevalence ratio was not significantly higher than that in populations without relevant comorbidities or risk factors [12].

Characteristics and risk factors

Intracranial aneurysms associated with pituitary adenomas can be classified by their spatial relationship to the tumor: non-adjacent, adjacent, or intra-adenoma. In non-adjacent cases, the aneurysm is separate from the adenoma and does not contact it, with intervening tissue between them. In adjacent cases, the aneurysm is in close contact with the adenoma, while the tumor capsule remains intact. In intra-adenomal cases, the aneurysm is wholly or partially embedded within the adenoma. Most aneurysms coexisting with pituitary adenomas are located outside the tumor [13]. Rarely, an intrasellar internal carotid artery aneurysm can be misdiagnosed as a pituitary macroadenoma and may be present clinically as pituitary apoplexy. Conversely, pituitary apoplexy with an intracerebral hemorrhage can mimic the rupture of an anterior-circulation aneurysm in a patient with a sellar lesion; the internal carotid artery and anterior communicating artery are most often involved [14].

Tumor-related factors are reported to be associated with intracranial aneurysms in patients with pituitary adenomas. In some series, growth hormone-secreting adenomas account for up to 50% of cases with coexisting aneurysms. Growth hormone excess may promote atherosclerotic and degenerative changes in the arterial wall of the circle of Willis, increasing susceptibility to aneurysm formation [11]. Although prolactinomas are the most common functional pituitary tumors, their reported coexistence with intracranial aneurysms appears lower than that of other pituitary adenoma subtypes [15].

Possible mechanisms of association

Several mechanisms may contribute to aneurysm formation in this setting, including mechanical effects of the adenoma on adjacent vessels, local hemodynamic changes related to increased vascular demand, and hormonal influences. No single mechanism fully explains aneurysm development [13].

In pituitary adenomas, tumor growth may alter local vascular microanatomy by compressing or tractioning adjacent vessels [16]. These changes may alter cerebral blood flow and increase hemodynamic stress, thereby promoting aneurysm formation [2]. In this context, Oh et al. reported cavernous sinus invasion as the factor most strongly associated with intracranial aneurysms, with older age as an additional significant correlate [11].

Growth hormone and related growth factors may contribute to aneurysm formation [16]. The relatively high proportion of growth hormone-secreting pituitary adenomas among cases with coexisting intracranial aneurysms supports the hypothesis that chronic growth hormone hypersecretion plays a role in aneurysm development in functioning tumors [17,18]. In addition, larger aneurysms located adjacent to the tumor suggest a local effect of growth hormone or insulin-like growth factor 1 (IGF-1) on the cerebral arterial wall, potentially promoting aneurysm formation [19].

Accordingly, acromegaly is associated with a higher prevalence of intracranial aneurysms, particularly in men. In a prospective study of 153 patients with acromegaly, Manara et al. reported that growth hormone levels correlate with intracranial aneurysm presence and that aneurysms more often involve the internal carotid artery than in the general population (67.5% vs 23-42%), with more frequent intracavernous segment involvement (22.5% vs 2-9%). They suggest a neuroimaging evaluation of the cerebral circulation during follow-up [20].

Following pituitary surgery, iatrogenic, traumatic, or mycotic aneurysms may occur as complications. Tsuchida et al. describe subarachnoid hemorrhage during transsphenoidal resection of a nonfunctioning macroadenoma. They report that, after most of the tumor was removed, the tumor capsule abruptly collapsed, potentially exerting traction on an anterior communicating artery aneurysm. The aneurysm was small and was not identified on preoperative bilateral carotid angiography, raising the possibility that it developed intraoperatively. Similar to our case, the patient did not regain consciousness after surgery; the diagnosis was made on postoperative computed tomography, and management required ventricular drainage and aneurysm clipping, with a favorable clinical outcome [21].

Finally, radiation therapy may also contribute to increased aneurysm risk. In 2011, Endo et al. reported a patient who developed subarachnoid hemorrhage and epistaxis due to rupture of an intrapetrous internal carotid artery aneurysm after prior radiation therapy and transsphenoidal surgery. They describe skull base bony changes related to previous surgery, radiation, and tumor invasion, and suggest that these factors may explain the unusual combination of subarachnoid hemorrhage and epistaxis after rupture of a petrous-segment internal carotid artery aneurysm [22].

Imaging study

No evidence-based guidelines support routine screening for asymptomatic intracranial aneurysms in all patients undergoing preoperative assessment for pituitary adenomas. However, although postoperative subarachnoid hemorrhage and significant vascular injury are uncommon, they can be catastrophic and warrant risk assessment [6]. Detailed preoperative anatomical assessment remains the most reliable strategy to reduce complications of transsphenoidal surgery, particularly vascular injury [22].

Magnetic resonance imaging (MRI), given its ability to delineate vascular anatomy, is the preferred modality for preoperative evaluation of pituitary tumors. A study assessing intracranial aneurysm detection with MR angiography reports a sensitivity of 95% (95% CI, 89-98%) and a specificity of 89% (95% CI, 80-95%) [23]. In preoperative MRI, particularly in recurrent pituitary adenomas, where fibrosis may distort normal anatomical relationships, careful review is required to identify newly formed aneurysms [24].

When tumor expansion or cavernous sinus displacement is suspected, preoperative angiographic evaluation, conventional angiography, or magnetic resonance angiography is often performed. Likewise, if MRI suggests vascular abnormalities or altered flow, particularly in the anterior circulation, angiographic imaging should be considered to exclude a coexisting aneurysm [16,22].

Management

The presence of an intracranial aneurysm within a pituitary adenoma or in the setting of pituitary apoplexy can alter the surgical approach by increasing the risk of intraoperative rupture and subarachnoid hemorrhage, which may adversely affect the patient’s outcome despite an otherwise benign surgical pathology.

The rupture of an undiagnosed aneurysm during elective transsphenoidal surgery is a severe complication, as demonstrated by the presented clinical case. Limited visualization of the bleeding source and rapid blood accumulation within the operative field restrict effective hemorrhage control, resulting in substantial blood loss and the need for immediate hemodynamic support until definitive hemostasis is achieved [24]. Although well described in the literature, this event is fortunately rare. While the mechanism of rupture remains debated, intraoperative maneuvers used to improve exposure, such as air or saline instillation via a lumbar drain, bilateral jugular venous compression, and the application of positive end-expiratory pressure, have been associated with perioperative bleeding, potentially through direct mechanical effects or hemodynamic perturbations [6].

During transsphenoidal surgery, the appearance of a blue-violet discoloration beneath the typically transparent sellar dura should raise suspicion of a potential vascular abnormality, such as an aneurysm [24]. In these cases, ultrasound with micro-Doppler examination is crucial before proceeding with a dural incision.

Finally, the presence of an intracranial aneurysm requires accurate localization to enable safe resection of a pituitary adenoma [14]. When both lesions coexist, a single-stage approach is rarely necessary; however, if both pathologies can be addressed through the same craniotomy, this may be an option. When urgent pituitary surgery is not required, aneurysm treatment, open or endovascular, depending on location, size, and patient factors, should be performed first, followed by transsphenoidal resection of the pituitary adenoma as a second-stage procedure.

## Conclusions

Although the exact incidence and mechanisms underlying the association between pituitary tumors and intracranial aneurysms are still not resolved, the challenges in diagnosis and the implications for treatment of this relatively rare coexistence are significant. As a result, a vascular lesion is possible in all patients with parasellar lesions. The use of cerebrovascular imaging before the surgical management of pituitary adenomas remains debatable and requires further investigation. MRI and angiography provide crucial information in cases where intracranial aneurysms coexist with pituitary tumors, with the treatment of the aneurysm taking precedence over that of the pituitary tumor.

The debate regarding indications for imaging and its choice may persist until stronger evidence emerges. However, current data support a pragmatic approach: in contemporary practice, critical diagnostic imaging should not be delayed or omitted. Although broader preoperative imaging may increase upfront costs, the clinical and economic consequences of an undetected aneurysm, particularly if rupture occurs, are greater, as illustrated by the fatal outcome in our case. In response, our institution has adopted routine preoperative computed tomography angiography for patients undergoing pituitary surgery. Even though this complication is uncommon, it is well described; identifying aneurysms preoperatively enables appropriate planning and helps ensure timely, coordinated management.
